# Opium in Metro-Peritonitis

**Published:** 1871-04

**Authors:** W. L. Johnson

**Affiliations:** Crawfordsville, Indiana


ARTICLE VII.—*Opium in Metro-Peritonitis. Review of a Criticism.* By W. L. JOHNSON, M.D., Crawfordsville, Indiana.

An article in the February number of the *Chicago Med. Journal*, entitled Opium in Metro-Peritonitis, by Theodore Griffin, M.D., with comments by Walter Hay, one of the editors of the *Journal*, was read by me with some surprise; surprised that such a criticism should come from such a source.

The subscribers of the *Chicago Med. Journal*, are of the greater part former students of Rush Med. College, and the *Journal* is understood to be the organ of, and to reflect the principles and teachings of that institution. As I left that college but three years ago, its teachings and precepts are still fresh in my mind. Allen—although usually opposed to the heroic plan of treatment of disease—says, in giving opium or other anodynes, the object is to always relieve pain, and that excessive pain must always be relieved; the practitioner who does not accomplish this, does not his duty. Gunn says opium has no dose—in excessive pain there is no dose for opium. I am not positive as to his exact words, but his teachings are the same as Doctor Griffin's in his article—*let the indications be met in the treatment*, *howsoever large the dose of the drug may be.* I hold that this is the doctrine and practice of every member of the faculty of Rush Med. College. If there is an exception it should be made known, as it is a matter of no little importance. It is important to know what is good practice in the administration of opium—whether the practitioner is justified in giving three or four grains every half hour when there are excessive and lancinating pains; whether it is right to give five grains each hour for a day, or sometimes in extreme cases even to give tincture of opium in one dram doses every fifteen minutes until pain is relieved. Let Prof. Allen answer to this point, for himself, for Prof. Miller, for Prof. Ingals, for Prof. Gunn, for all the faculty of his college. According to my understanding of the teachings and opinions of these gentlemen, they would justify Doctor Griffin in his treatment of the case reported.

A sentence, within the criticism enclosed in brackets, followed by an initial A, would lead the reader to suppose that Prof. Allen endorsed the whole criticism. But I am inclined to believe Prof. Allen did not carefully read the article and criticism. I consider the criticism extremely lame.

Let us, if you please, give this criticism a short review.

The article is "published as an illustration of the degree to which the human system can be educated into the frightful habit of opium eating." This same idea is enunciated in one of the four possible explanations given at the end of the criticism. "The woman was an opium eater already." The absurdity of this idea is proved by the fact that the woman was narcotized by the last six grains, after the pain had abated, when she had been taking such large doses for so long a time without any signs of opium poisoning, remembering at the same time that there was an interval of sleep just preceding the poisonous doses, so that the effect cannot be attributed to any of the drug taken while there was great pain. It was just as all of us were taught at Rush Med. College and as experience has shown us in practice since. Opium is an antidote of pain, and, *vice versa*, excessive pain requires excessive doses of opium.

Modern physiology surely does not teach that when the uterus and surrounding peritoneum are inflamed and the "bowels are constipated," "pulse 90 and bounding," that the use of opium is contra-indicated. Physiology does teach that inflammation of the parts mentioned can be controlled to some extent by the influence opium has on the nervous system, through the blood, be the bowels constipated or not. What has that portable privy, the rectum, to do with an inflamed uterus and peritoneum? A brisk purgation might be well to lessen the force of the circulation, but is of small importance compared with the influence to be obtained by large doses of opium. There are some quibbles as to words used and the author's style of expression, which properly lie between the author and his critic. Let us pass them.

There seems to me nothing strange in the report of the first day's attack, when the "symptoms yielded to a little mild treatment," or that there should be a relapse on the next day so severe as not to yield to large doses of opium. There might be a quibble raised as to the way the word *symptoms* is used, but that is a small affair.

The critic follows the phrase "no signs of opium poisoning?" with a note of interrogation; and gives some quotations as if to prove that there was opium poisoning, or at least the use of opium contra-indicated.

"The nervous system much disturbed and agitated," as one quotation—this I hold to be the effect of the disease, and requiring an increased dose of the drug. "No urine passed for 11½ hours"—which proves nothing unless a partial paralysis of the bladder, which the opium would not aggravate but rather tend to relieve. "The mammary secretion gone"—this should not affect the general treatment.

And although at length the "nervous symptoms were better," "with some subsultus tendinum," yet the author is justified in maintaining his previously large doses; first, because there being subsultus tendinum proves the change for the better not great, and second, because after the continued use of opium for many hours, it requires larger doses, to get the same effect, than when first administered. It is a familiar rule in therapeutics—a continued use of opium requires an increase in the doses. Six grains an hour now, even with the symptoms a little better, have no more effect than four grains thirty hours ago, even with symptoms worse.

Notice, farther on, how severe the critic can be. "Was she better? More opium! Was she unchanged? More opium!! Was she still worse? still more opium!!!" A new exclamation point at each question and answer, and the way he has written it there does seem to be some mirabile dictu about it. But let us reverse these explosions of words and see how they sound. "Was she worse? more opium,"—well, why not? If it is the onward march of the disease and the lancinating pains are worse and the nervous system more disturbed and agitated, it surely would not be well to decrease a dose which had not acted too much or too well in its controlling influence. The veriest neophyte would say, increase the dose—work carefully, but increase the dose. "Was she unchanged? increase the dose; more opium." To be sure, and for the same reason as when getting worse. "Was she better? more opium." If after the continuous use of this drug for 38 hours and symptoms are but slightly ameliorated, it is still necessary to keep up the increase of dose. Who says nay?

There is a criticism as to the delay in the use of the catheter. Sixteen hours is no great length of time for urine to remain in the bladder. It may have been too long a time in this case. He was the best judge who was there to see.

As to the "arrest of the renal secretion," mentioned at one time, it seems to be altogether an invention of the critic, as the author mentions nothing but retention of urine—no suppression. Suppression is attended with uremic poisoning—and there was none in this case.

It would seem that the editor has some spite or jealousy to work against this author by the way plain facts in this case are perverted and misstated. The criticism surely has not the foundation of good sound knowledge either theoretical or practical, not but the editor is learned, but in this case he has in some inexplicable manner been led astray.

---

When, at the suggestion of a professional friend, whose large experience entitled his opinions upon kindred subjects to respect, we entered our protest upon the pages of this JOURNAL, (Feb. No.) against the reckless use of a certain valuable remedial agent, we certainly had no expectation of seeing the necessity for the protest so speedily and fully demonstrated as in the above communication, which speaks for itself and needs no "Review." On our own behalf we shall refer to but one point in this *gentlemanly* communication, *i. e.*, to the charge of personal spite and malice; to which our defense is, that we were first made aware of the existence of the author, by the publication of the "Report" in question. In view of the thorough self-condemnation of the above, we publish it entire, in all its native elegance of style and diction, and courtesy of tone.

As to the impression which seems to rest upon the mind of our courteous correspondent, that the JOURNAL is the organ of the

Rush Medical College, it is, like some other of his impressions, a delusion, which not having created we are not responsible for. Concerning the teachings of the Professors of that Institution as a body, knowing nothing, we have nothing to say.

Professor Miller particularly desires that the misrepresentation of his views contained in the above, be corrected, assuring us that he had consistently and persistently warned his pupils against the pernicious theories thus falsely attributed to him; that he had authorized the use of opium only to allay pain and to retard the retrograde metamorphosis of tissue.

Prof. Gunn informs us that while he is quoted, with verbal accuracy, as refusing (of course) to specify doses of opium—he declines the paternity of such wholesale therapeutics.

Had our correspondent, by the assumption of a somewhat more courteous tone, entitled himself to courtesy in turn, we might have placed him in possession of further information concerning the "case" which would have enlightened even his understanding, but he must e'en bore his own hole through the mill-stone, or we fear he will never see the "other side."

We trust that no patient may die under the heroic system advocated above, lest the officiating executioner might perchance receive the baptism of Gratiano—of hemp, with twelve god-fathers. W. H.

---

***Spontaneous Combustion.***

Facts accumulating with reference to this subject seem to show that a much greater number of articles in common use may spontaneously ignite, than was formerly supposed. An instance of this kind has lately occurred in which oily iron chips, or borings from a gun factory, upon being carried out and exposed to the air, oxydized so rapidly that the oil, with which they were saturated, burst into a fierce flame. In general, it may be stated that it is dangerous to leave any material of a porous or spongy nature, saturated with oily matter, in a position where it can communicate fire to other combustible materials.—*Med. Jour.*

